# *Escherichia coli* Remodels the Chemotaxis Pathway for Swarming

**DOI:** 10.1128/mBio.00316-19

**Published:** 2019-03-19

**Authors:** Jonathan D. Partridge, Nguyen T. Q. Nhu, Yann S. Dufour, Rasika M. Harshey

**Affiliations:** a Department of Molecular Biosciences, University of Texas at Austin, Austin, Texas, USA; b Department of Microbiology and Molecular Genetics, Michigan State University, East Lansing, Michigan, USA; The Ohio State University

**Keywords:** CheZ, chemotaxis, flagellar motility, Lévy walk, swarming

## Abstract

Many flagellated bacteria “swarm” over a solid surface as a dense consortium. In different bacteria, swarming is facilitated by several alterations such as those corresponding to increased flagellum numbers, special stator proteins, or secreted surfactants. We report here a change in the chemosensory physiology of swarming Escherichia coli which alters its normal “run tumble” bias. E. coli bacteria taken from a swarm exhibit more highly extended runs (low tumble bias) and higher speeds than E. coli bacteria swimming individually in a liquid medium. The stability of the signaling protein CheZ is higher in swarmers, consistent with the observed elevation of CheZ levels and with the low tumble bias. We show that the tumble bias displayed by wild-type swarmers is the optimal bias for maximizing swarm expansion. In assays performed in liquid, swarm cells have reduced chemotactic performance. This behavior is specific to swarming, is not specific to growth on surfaces, and persists for a generation. Therefore, the chemotaxis signaling pathway is reprogrammed for swarming.

## Abstract (precis)

The fundamental motile behavior of E. coli is a random walk, where straight “runs” are punctuated by “tumbles.” This behavior, conferred by the chemotaxis signaling system, is used to track chemical gradients in liquid. Our study results show that when migrating collectively on surfaces, E. coli modifies its chemosensory physiology to decrease its tumble bias (and hence to increase run durations) by post-transcriptional changes that alter the levels of a key signaling protein. We speculate that the low tumble bias may contribute to the observed Lévy walk (LW) trajectories within the swarm, where run durations have a power law distribution. In animals, LW patterns are hypothesized to maximize searches in unpredictable environments. Swarming bacteria face several challenges while moving collectively over a surface—maintaining cohesion, overcoming constraints imposed by a physical substrate, searching for nutrients as a group, and surviving lethal levels of antimicrobials. The altered chemosensory behavior that we describe in this report may help with these challenges.

## INTRODUCTION

Swarming is a group phenomenon widespread among flagellated bacteria wherein the bacteria migrate collectively over a solid surface and display increased resistance to antimicrobials (1–6). The strategies for effective “swarm” movement are as varied as the bacteria themselves (1, 3, 7–9), but a feature common to all swarms is the continual swirling motion of millions of densely packed bacteria steadily pushing the swarm outward, acquiring more and more surface territory (5). The fact that so many bacterial species display this form of motility argues that swarming is an important means of migration and survival in the bacterium’s natural habitats.

Swarming bacteria have been classified into two categories: robust swarmers that can navigate across any agar surface, particularly across hard agar (1.5% agar and above), and temperate swarmers that can swarm only on a softer agar surface (0.5% to 0.8% agar) (1, 3, 5). The swarmers in the latter category were first discovered in Serratia marcescens (10) and were observed later in Escherichia coli and Salmonella enterica (11), Bacillus subtilis (12), Pseudomonas aeruginosa (13), and *Yersinia* species (14), among others (1, 3). Movement over an agar surface presents several challenges not encountered during swimming such as those associated with surface tension, friction, and hydration (5, 15). Bacteria have evolved several adaptations to overcome these challenges, including secretion of surfactants, increasing flagellum numbers, and deploying special stators or stator-associated proteins, as well as modulating the expression of several genes (3, 5). The characteristics of swarming motion of a subset of the temperate swarmers—E. coli, S. enterica, B. subtilis, and S. marcescens—have been analyzed in several studies and, despite these bacteria belonging to different phyla, appear to be remarkably similar (16–21). The common features of this motion can be summarized as follows. A swarming colony is dense and multilayered in the interior where the swirling motion is the most intense. Here, dynamic packs or groups of cells continuously stream in different directions, exchanging bacteria between streams. The advancing edge of the swarm is generally monolayered. Here, cells move more slowly and often pause and then move back toward the center of the swarm. The speeds of movement of individual bacteria within fast-moving streams are reported to be comparable to the swimming speeds seen in bulk liquid (16, 22), as are their flagellar mechanics (23). The motion of the bacteria within the swarm is superdiffusive, consistent with a Lévy walk (LW) model (19), which is a continuous-time random walk in which particles move with a fixed speed, making sharp turns at time intervals that follow a power law distribution (24, 25). Cells in the swarm do not strictly follow the collective fluid flow and may move in a direction perpendicular to or even against it, generating trajectories different from those seen in the crowd (21).

**FIG 1:**
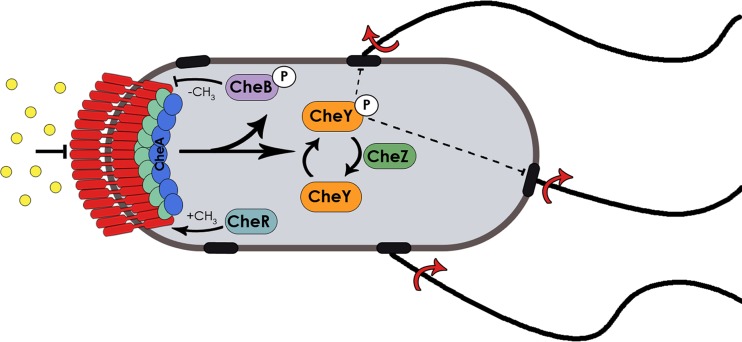
The E. coli chemotaxis system and its control of motility. Extracellular ligands (circles) are detected by transmembrane chemoreceptors (MCPs; red) connected to a cytoplasmic kinase (CheA; blue) via a linker (CheW; green). A basal rate of autophosphorylation generates CheA∼P, which transfers its phosphate to two response regulators (CheB and CheY). CheY∼P binds to the flagellar motor, reversing its default counter-clockwise (CCW) rotation to clockwise (CW). CCW states promote runs, while CW states promote tumbles. The phosphatase CheZ dephosphorylates CheY∼P, terminating the tumble response. In the absence of chemoeffector gradients, the cells swim in a random walk of runs and tumbles. Chemoattractant binding represses CheA kinase activity, decreasing tumbling probability and extending runs and allowing cells to move up an attractant gradient. CheB∼P governs the adaptation response that returns the MCPs to their presignaling state by removing methyl groups added to the MCPs by the methyltransferase CheR, so that the MCPs remain responsive to changes in the chemoeffector concentration. MCP methylation controls their kinase on-off states.

Swimming motility in bulk liquid is best studied in E. coli, which has several flagella in a peritrichous arrangement. Individual bacteria swim by rotating the helical flagellar filaments, each driven at its base by a bidirectional rotary motor (26–28). The default direction of motor rotation is counter-clockwise (CCW), which propels the cell forward in a “run.” When one or more motors turn clockwise (CW), the cell randomly changes direction or “tumbles.” In isotropic chemical environments, E. coli swims in a random walk by interrupting runs with tumbles. Swimming cells perform chemotaxis by extending their runs in the direction of chemical gradients using an elaborate signaling system that controls the flagellar motor switching frequency (Fig. 1). The signaling pathway adapts to constant stimuli to allow the system to function in a wide range of signal concentrations (29). Transmembrane chemoreceptors, known also as methyl-accepting chemotaxis proteins (MCPs), bind specific chemical ligands in the periplasm. A conserved set of cytoplasmic signaling proteins control flagellar rotation and sensory adaptation. CheW forms a scaffold with the receptors to control the kinase activity of CheA, which in turn phosphorylates CheY. The phosphorylated form of CheY (CheY~P) interacts with the flagellar motor to promote switching of the flagellar motor to CW rotation. CheZ rapidly dephosphorylates CheY~P to maintain a fast response to signals. The slow adaptation of the receptor activity is mediated by CheB and CheR, which de/methylate the MCPs to return CheA activity to its basal level. In the absence of chemoeffector gradients, E. coli has a basal tumble bias due to a basal rate of CheA autophosphorylation.

When swimming in chemoeffector gradients, the cells respond to an increase in attractant concentration by suppressing CheA kinase activity, resulting in longer runs in favorable directions (Fig. 1). The basal rate of motor switching, or tumble bias, in the absence of gradients is determined by the activity of CheA relative to CheZ. Basal CheA activity is determined by the relative activities of CheR and CheB through receptor methylation. Therefore, the relative levels of expression of different proteins in the chemotaxis pathway determine the basal behavior of motile cells (30, 31).

We show in this report that E. coli swarms alter their physiology by increasing the levels of the CheZ phosphatase relative to those of other proteins in the chemotaxis pathway. This change in a key signaling protein is consistent with the low level of tumble bias observed, which is apparently optimal for swarm expansion and may confer other benefits for survival of the group.

## RESULTS

### E. coli reduces tumbling frequency and increases swimming speed during swarming.

It has long been observed by our laboratory that cells taken from a swarm—whether E. coli, *Salmonella*, or S. marcescens—swim smoothly upon transfer to liquid media (cited as unpublished data in reference 1), presenting a contrast to the run/tumble behavior of cells cultivated in liquid media. To quantify this behavior, we tracked the motion of individual E. coli cells lifted from the swarm into liquid. Cell trajectories were monitored in a pseudo-two-dimensional (2D) channel between a glass slide and coverslip ∼10 μm deep. The basal behavior of cells was tracked for 100 s in an isotropic environment at room temperature, and the trajectories were analyzed as previously described (31). In preliminary experiments, different regions of the swarm were sampled (see Fig. S1A in the supplemental material). Even genetically identical cells display a continuous distribution of phenotypes due to cell-to-cell variability in chemotaxis protein numbers (31–33), consistent with the observed distribution of various motility parameters quantified in Fig. S1. Cells with a diffusion coefficient of less than 10 μm^2^/s are driven only by Brownian motion and were classified as non-motile and not included in the analyses of swimming speed and tumble bias (Fig. S1B). Little difference in the measured motility parameters was observed in cells taken from different regions of the swarm. Detailed investigations were conducted solely from cells at the advancing swarm edge, which are expected to not be depleted of nutrients.

10.1128/mBio.00316-19.1 FIG S1 Preliminary experiments monitoring the tumble bias of E. coli swarm cells from different regions of the swarm. (A) Sampling positions across the swarm, red dot denotes inoculation point. (B and C) Swimming speed (in micrometers per second) and tumble bias of motile swarmer cells, respectively, for cells collected from positions A and B on the swarm plate. Each distribution was calculated from more than 600 individual trajectories (400 min cumulative time) combined from each of two independent experiments. Mean, circle; median, square. Download FIG S1, TIF file, 2.2 MB. Copyright © 2019 Partridge et al. 2019 Partridge et al. This content is distributed under the terms of the Creative Commons Attribution 4.0 International license.

**FIG 2:**
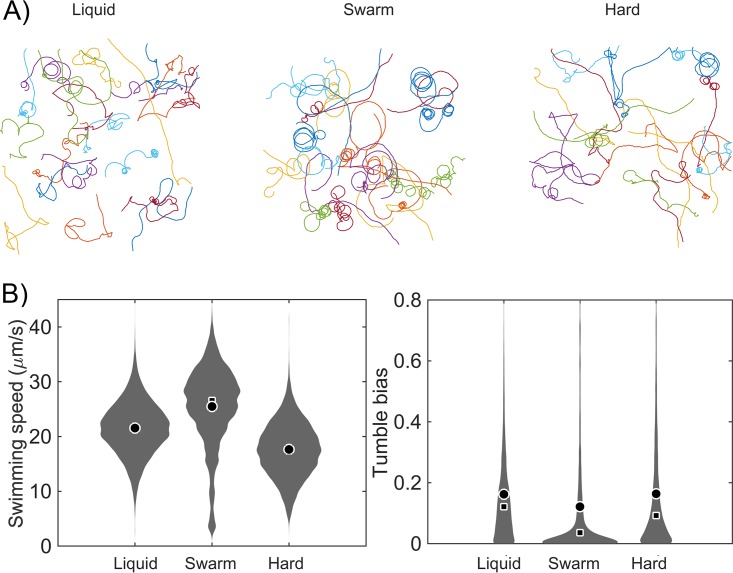
Behavior of E. coli cells in a pseudo-2D environment. Cells were grown in LB (liquid), LB swarm agar, or LB hard agar before transfer to LB liquid for observation (labeled “Liquid,” “Swarm,” or “Hard,” respectively). Cell movement was recorded for 100 s using phase-contrast microscopy at 10× magnification. (A) Trajectories of single experiments. Different colors correspond to individual tracks. B) Probability distribution of swimming speeds (indicated in micrometers per second) (left) and cell tumble biases (right). Each distribution was calculated from more than 5,000 individual trajectories (>1,000 min of cumulative time) for each condition combined from nine, eight, and five independent experiments for liquid medium, swarm agar, and hard agar conditions, respectively. Mean, circle; median, square. Separate experimental data sets can be seen in Fig. S2A.

In-depth comparisons were made between cells harvested from the swarm edge and those grown in liquid culture, both of which were expected to be in a similar growth phase (34). Representative cell trajectories from each condition showed distinctive behaviors (Fig. 2A). Liquid-grown cells showed typical run-tumble behavior (31), while cells transitioning from the swarm had longer runs and fewer tumbles. A quantitative analysis of the population tumble biases shows a shift of the median from ∼0.12 in liquid to ∼0.04 from the swarm edge (analysis of variance [ANOVA] *P* value < 10^−69^) (Fig. 2B; see also Fig. S2). The majority of cells from the swarm had a very low tumble bias. The swimming speed of the liquid-grown cells was distributed around 21 μm/s, whereas most swarmers swam around 25 μm/s (ANOVA *P* value < 10^−100^) (Fig. 2). The response was specific to swarm agar, as it was abolished by increasing agar hardness, a condition that does not support swarming in E. coli (Fig. 2; see also Fig. S2) (1.5% [wt/vol] agar, labeled “Hard”). Thus, the behavior of cells in the swarm appears to represent something more than a response to growth on a solid surface.

10.1128/mBio.00316-19.2 FIG S2 Swimming speed and tumble bias of E. coli under various growth conditions. (A to C) Distributions of cell speeds (left) and tumble bias (right) of cells grown in LB broth (A) or swarm agar (B) or hard agar (C) before being transferred to LB. The distributions were calculated from more than 5,000 individual trajectories (>2,400 min cumulative time) from 9 independent experiments for liquid-grown cells (A), 8 experiments for swarmer cells (B), and 5 experiments for cells cultivated on hard agar (C). Plots averaging the data from these replicates are presented in Fig. 2. Mean, circle; median, square. Download FIG S2, TIF file, 0.8 MB. Copyright © 2019 Partridge et al. 2019 Partridge et al. This content is distributed under the terms of the Creative Commons Attribution 4.0 International license.

**FIG 3:**
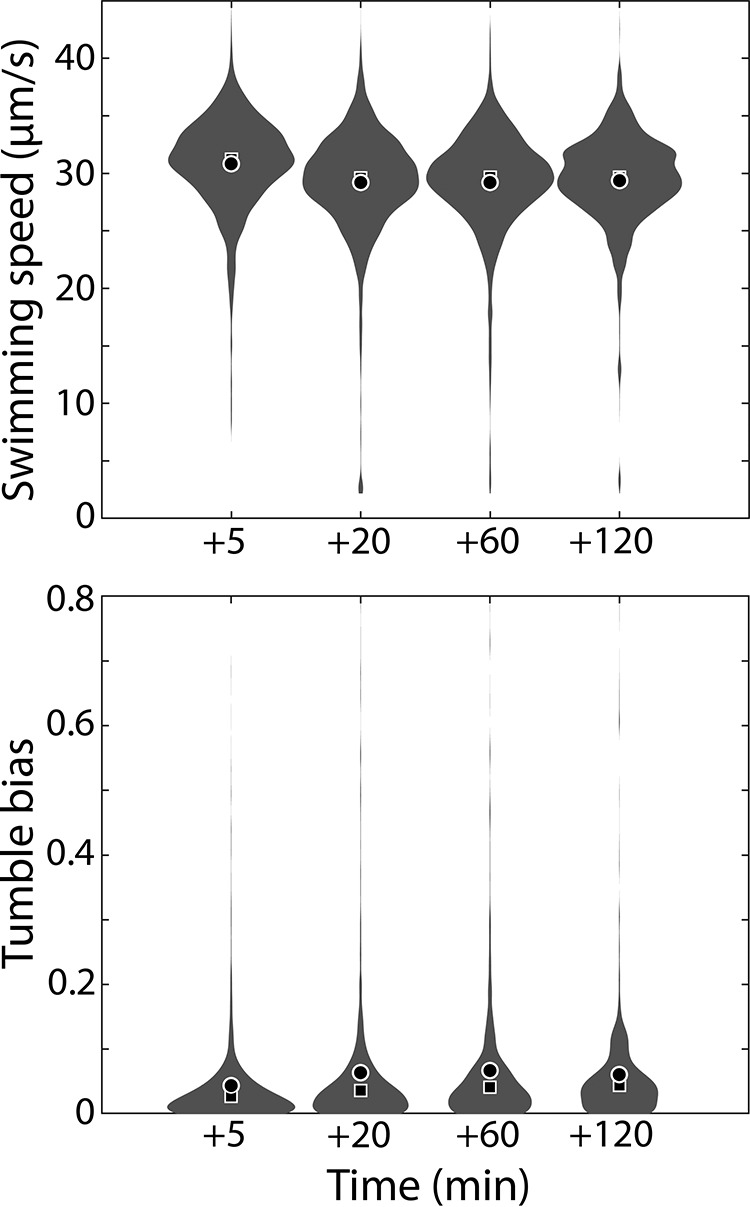
Swim speed and tumble bias distribution of E. coli swarmers after prolonged resuspension in liquid. Probability distribution data representing swimming speed (top, µm/s) and cell tumble biases (bottom) are shown. Cells grown on swarm agar and transferred to LB were monitored over 120 min. Each distribution was calculated from 600 to 6,000 individual trajectories (200 to 1,300 min cumulative time) combined from at least two independent experiments, respectively. The discrepancy in swimming speeds between Fig. 2 and 3 is likely due to differences in the number of experimental replicates (see Fig. S2 for examples). Mean, circle; median, square.

The low level of tumble bias was unlikely the result of the chemotactic response during transfer from the swarm to liquid because cells from the swarm edge should not have experienced low chemoattractant concentrations and, even if they had, should have adapted during the 15 to 20 min required to prepare the swarm cells for observation (see Materials and Methods) (35–38). Indeed, during intermittent observation of the swarm cells after transfer into liquid Lennox broth (LB), the tumble bias gradually increased but remained lower than the tumble bias of cells grown in liquid after 120 min at room temperature (ANOVA *P* value < 10^−23^) (Fig. 3). Measurement of cell counts showed that cells went through one division by 120 min (Fig. S3), consistent with “dilution” of the altered tumble bias phenotype of swarm cells through division. Low tumble frequencies were also recently reported for E. coli swarm cells tracked in a 3D tracking device after several washing steps (22). The increase in swimming speed of swarmers can be partially explained by the observed correlation between the mean speed and the level of tumble bias in single cells (Fig. S4). Even though inertia is negligible at the bacterial scale, it takes ∼0.5 s for a cell to recover full swimming speed after a tumble (39).

10.1128/mBio.00316-19.3 FIG S3 Kinetics of cell growth under the experimental conditions used for tracking. Cells from swarm agar were transferred to LB glucose (0.5% [wt/vol]) as described for the experiment represented in the top panels of Fig. 3, held for 5 to 120 min at room temperature, and sampled at these times for CFU counts on LB hard agar. Cell numbers had doubled by 120 min. The data are representative of results from three biological replicates, each tested in triplicate, and are presented as log2 values with error bars indicating standard deviations of the means. Download FIG S3, TIF file, 0.1 MB. Copyright © 2019 Partridge et al. 2019 Partridge et al. This content is distributed under the terms of the Creative Commons Attribution 4.0 International license.

10.1128/mBio.00316-19.4 FIG S4 Density plot of single-cell mean swimming speed as a function of tumble bias. The density data were generated from 75,000 cell trajectories compiled from the experiments described in this work. Download FIG S4, TIF file, 0.1 MB. Copyright © 2019 Partridge et al. 2019 Partridge et al. This content is distributed under the terms of the Creative Commons Attribution 4.0 International license.

Overall, the results support the hypotheses that swarmer cells have an inherently low tumble bias and higher run speed than cells grown in liquid media and that the behavior persists for longer than the time scale of adaptation of the chemotactic response, suggesting that E. coli has a programmed behavioral response to facilitate swarming.

### Single motors of swarmers show reduced switching and higher speeds/torque.

**FIG 4:**
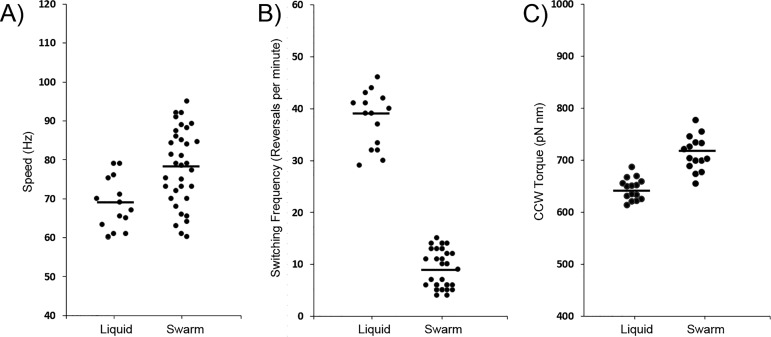
Behavior of single motors of E. coli over 60 s. Rotation of motors of E. coli MG1655 *fliC* pFD313 (expressing FliC*^st^*), taken from broth-grown cultures (liquid) or picked from the surface of swarm agar, was monitored by recording the motion of 0.75-µm-diameter polystyrene beads attached to sheared “sticky” filaments. Data representing rotational speeds (Hz) (A), switching frequency (reversals per minute) (B), and torque (C) are shown for 15 motors of liquid-grown cells and for 35 swarmer motors. Torque (indicated in piconewtons per nanometer) was derived from CCW speeds for 15 motors determined under both conditions. The black bar represents the mean value, with *P* values of <0.05 for panels A and C and <0.01 for panel B. Examples of individual motor traces are shown in Fig. S5.

To corroborate the results obtained from single-cell tracking with the behavior of single flagellar motors, we monitored the rotation of polystyrene beads attached to “sticky” flagellar filament stubs (FliC*^st^*) (40) on tethered cells in the absence of chemotactic stimuli (Fig. 4; see legend for *P* values). E. coli cultivated in liquid showed average speeds of 68 Hz ± 7 Hz (Fig. 4A) and 38 ± 5 reversals per min (rv.p.m.) (Fig. 4B), values that are similar to those that we reported previously (41) (15 motors captured). It was difficult to get swarm cells to stick to the glass slide, likely due to an altered outer membrane composition (42); nevertheless, we recorded the behavior of 35 swarmer motors. All showed a strong CCW bias, with reversals averaging 9 ± 4 per min (Fig. 4B). Their speeds averaged 78 ± 10 Hz, higher than the speeds seen with motors of cells grown in liquid. While several individual motors of swarmers showed speeds similar to that of the liquid-grown cells (Fig. 4A; see also Fig. S5 [compare liquid and swarm 1]), around half the population showed much higher speeds (Fig. 4A; see also Fig. S5 [compare liquid and swarm 2]). Torque was calculated from the measured CCW motor speed and the estimated drag exerted on the microbead attached to the flagellar stub as described earlier (43). The torque of the flagellar motor increases in swarmers by 10% to 712 ± 33 pN compared to 644 ± 21 pN in swimmers (Fig. 4C).

10.1128/mBio.00316-19.5 FIG S5 Behavior of single motors of E. coli over 60 s. Representative motor traces from a liquid-grown cell and two swarmer cells are indicated. The Swarmer 1 and 2 traces are representative of the two speed populations observed (see Fig. 4). The positive and negative values represent CW and CCW rotations, respectively. Switching (reversal in motor direction) occurs when the trace crosses zero. Download FIG S5, TIF file, 0.4 MB. Copyright © 2019 Partridge et al. 2019 Partridge et al. This content is distributed under the terms of the Creative Commons Attribution 4.0 International license.

In summary, the single-motor data are consistent with the tracking data in showing that swarmers inherently have a low tumble bias, higher motor speed, and higher motor torque.

### Swarmers migrate poorly in chemoeffector gradients.

**FIG 5:**
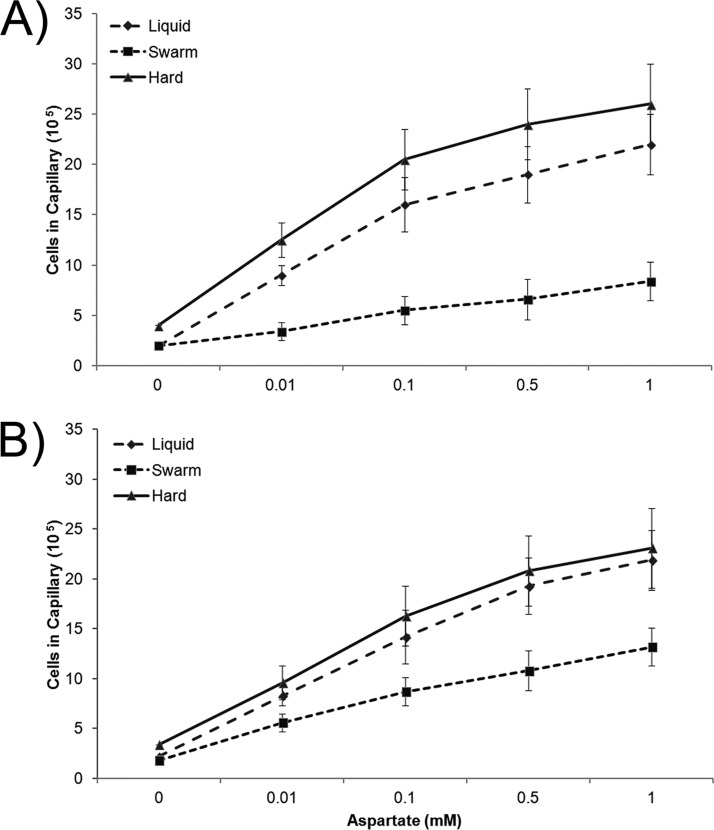
Quantification of chemotactic performance of E. coli cultivated under liquid, swarming agar, and hard agar conditions. Response to aspartate was measured using capillary assays as described in Materials and Methods. (A) Cells assayed immediately after harvesting. (B) Cells allowed an extra 45-min incubation after collection. Cell numbers are derived from three biological replicates, each checked in triplicate. Error bars represent standard deviations from the means.

To test the chemotactic performance of swarmers in liquid, we employed the classic capillary assay pioneered by Adler and Mesibov (44, 45) (see Materials and Methods). The results confirmed that cells transferred from a swarm to chemotaxis buffer (CB) were less able to migrate into a capillary filled with the attractant aspartate than those grown in liquid media (Fig. 5A). As a control, to ensure that this response was not due to growth on a surface, cells grown on a hard agar surface were compared alongside and observed to be uncompromised in their ability to respond to the chemoattractant, showing a slightly better response than the liquid-grown cells (Fig. 5A). To test the duration of the altered chemotaxis response, cells from both swarm agar and hard agar were held in CB for a further 45 min after harvesting before being tested in the capillary assay. The response of cells growing on hard agar was now similar to that of liquid-grown cells, while that of swarmers improved somewhat but remained suboptimal (Fig. 5B).

Overall, these data confirm that cells growing in a swarming state have reduced performance in tracking chemical gradients in liquid compared to those grown in either liquid media or on hard agar and that this behavioral change persists for longer than the adaptation time scale of the chemotaxis system. The data suggest that swarmers have altered some component(s) of the chemotaxis signaling pathway.

### Swarmer cells have elevated relative CheZ levels.

**FIG 6:**
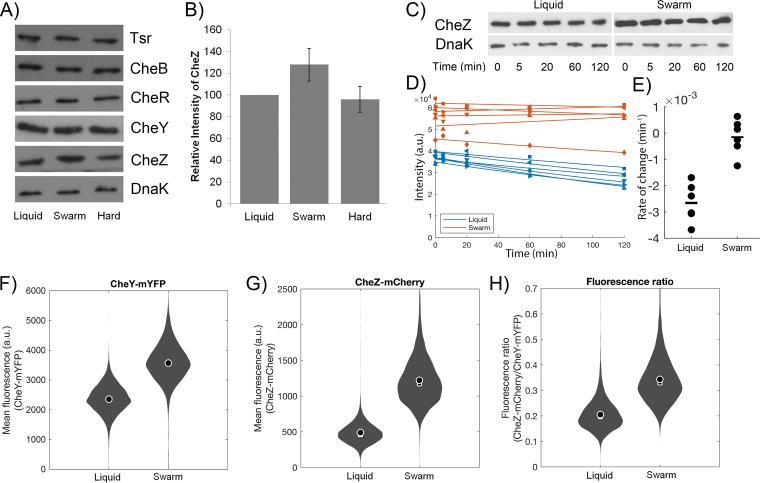
Changes in levels of key chemotaxis effector proteins under conditions of liquid, swarm agar, and hard agar growth. (A) Cells were harvested as described in Materials and Methods, and OD_600_ conditions were standardized before SDS-PAGE and subsequent immunoblotting versus Tsr, CheB, CheR, CheY, and CheZ antibodies were performed. DnaK antibody was used as a loading control and in the quantification of band intensities. Images are representative of three separate experiments, each analyzed in duplicate. (B) Quantitation of changes in CheZ levels across the three conditions—“Liquid,” “Swarm,” and “Hard.” Error bars represent standard deviations of the means, with *P* values for comparisons between conditions (liquid compared to swarm agar and liquid compared to hard agar) determined to be 0.041 and 0.044, respectively. (C) Representative Western blot showing a time course of degradation of CheZ (along with DnaK loading controls) from liquid and swarm agar after chloramphenicol treatment. (D) Kinetics of CheZ degradation after chloramphenicol treatment for six replicates each from liquid and swarm cells. a.u., arbitrary units. (E) Rate of CheZ degradation for liquid and swarm agar cell replicates. (F and G) Analysis of single-cell fluorescence to quantify the relative expression of CheY-mYFP (F) and CheZ-mCherry (G) with cells grown in liquid or from swarms. (H) Relative changes in the CheZ-mCherry/CheY-mYFP ratio between the liquid and swarm agar conditions. Each distribution represents an aggregate of data from three independent experiments (liquid, 7,600 cells; swarm agar, 10,400 cells). Mean, circle; median, square.

In swimming E. coli bacteria, Lévy walk behavior can be induced through stochastic fluctuations in the level of CheR, one of the key enzymes controlling CW behavior and adaptation in the chemotaxis signaling pathway (Fig. 1) (46). Given our observation that the swarmer cells had a low tumble bias, we surmised that these cells may have had altered levels of any of the following chemotaxis proteins that could alter the levels of CheY∼P, the tumble generator (Fig. 1): the major chemoreceptors Tsr and Tar, CheR, CheA, CheY, and/or CheZ. Previous microarray analysis had indicated that transcription of these genes was not altered during swarming (34). Therefore, we measured directly the levels of chemotaxis proteins for which we had antibodies by Western blot analysis. Levels of Tsr, CheB, CheR, and CheY showed no differences across liquid agar, swarm agar, and hard agar conditions (Fig. 6A). However, CheZ levels showed an increase of ∼30% in swarming conditions compared to liquid (Fig. 6B). To confirm our previous observation that there are no transcriptional changes in the flagellar genes during swarming in *Salmonella*, we performed real-time PCR (RT-PCR) analysis of the transcripts in E. coli. Probes for *tar, tsr, cheA, cheB, cheY, cheZ*, and *cheR* and a *gyrA* control were used (see Table S1 in the supplemental material). Transcript levels showed no significant increases under swarming agar and hard agar conditions compared to liquid-cultivated cells (Fig. S6). We conclude that CheZ levels are not elevated through transcriptional regulation.

10.1128/mBio.00316-19.6 FIG S6 RT-PCR data showing gene transcript changes in E. coli bacteria harvested under liquid agar, swarm agar, and hard agar conditions. Standardized liquid conditions are represented by a value of 1, with fold changes from cultivation on swarm agar or hard agar shown. Cultures were harvested in triplicate for each condition, with RT-PCR reactions for each carried out in duplicate. Results are normalized to the level of the *gyrA* transcript. Calculated *P* values were <0.05. Error bars represent standard deviations of the means. Download FIG S6, TIF file, 0.06 MB. Copyright © 2019 Partridge et al. 2019 Partridge et al. This content is distributed under the terms of the Creative Commons Attribution 4.0 International license.

10.1128/mBio.00316-19.10 TABLE S1 Oligonucleotide sequences of the primers used in this work. Download Table S1, DOCX file, 0.01 MB. Copyright © 2019 Partridge et al. 2019 Partridge et al. This content is distributed under the terms of the Creative Commons Attribution 4.0 International license.

Post-transcriptional mechanisms for altering protein levels may include increased mRNA translation and/or increased protein stability. Increased translation is unlikely to be the mechanism, because translation efficiency is normally dictated by the context of the Shine-Dalgarno (SD) sequence or codon usage (codon adaptation index [CAI]) (47). Examination of these parameters showed no major differences between the genes in this operon (*cheBRYZ*). To measure CheZ stability, we stopped protein translation by treatment of cells harvested from liquid and swarm plates with chloramphenicol and followed CheZ levels from 5 to 120 min, the range of time used when monitoring motility and chemotaxis behavior (Fig. 3 and 5). Western blots (Fig. 6C) confirmed that CheZ levels were ∼1.5× higher in swarmers (*P* value = 2.6 × 10^−4^) and that CheZ was stable for more than 6 h (mean degradation rates of 2.7 × 10^−3 ^min^−1^ in liquid and 1.5 × 10^−4 ^min^−1^ in the swarm) (Fig. 6D). The rates were significantly different between the two conditions (*P* value = 4.3 × 10^−3^) (Fig. 6E), suggesting that differential protein stability is likely the main factor explaining the difference in CheZ levels between swarm and liquid cultures.

To validate the population-average levels of CheZ (Fig. 6A and B) in single cells, we genetically constructed fluorescent protein (FP) fusions of CheY (CheY-mYFP [CheY-monomeric yellow fluorescent protein]) and CheZ (CheZ-mCherry) that were expressed from their native loci in the E. coli genome. The original clone did not spread as well as the wild type in soft-agar plates (0.3% agar), presumably because the fluorescent tags reduced the activities of the fusion proteins (48). We were able to restore the motility performance of the fluorescent strain to the wild-type level after three passages on soft-agar plates. The resultant strain, JLF394, had acquired a small deletion upstream of the promoter for the master flagellar regulon *flhDC*, expected to upregulate transcription (see Materials and Methods). We checked that the motile behavior of JLF394 in liquid and in the swarm was identical to that of wild-type E. coli by repeating the single-cell tracking experiments (Fig. S7A to C). Finally, we quantified the relative expression levels of CheY-mYFP and CheZ-mCherry in cells grown in liquid cultures by comparisons to cells sampled from the swarm using single-cell epifluorescence microscopy. The fluorescence signals formed foci consistent with the expected cellular localization of both CheY and CheZ (Fig. S7D and E) (48). The analysis of the mean total fluorescence from individual cells revealed that the levels of both CheY-mYFP and CheZ-mCherry had increased in the swarmers (ANOVA *P* value <10^−100^) (Fig. 6F and G). However, the relative levels of CheZ-mCherry to CheY-mYFP were higher in swarmers than in cells from liquid cultures (ANOVA *P* value <10^−100^) (Fig. 6H), consistent with the Western blot analysis and observed reduction in tumble bias in swarmers.

10.1128/mBio.00316-19.7 FIG S7 Behavior and protein expression from fluorescently labeled strain JLF394. (A and B) Swimming speed (ANOVA *P* value < 10^−23^) (A) and tumble bias distributions (ANOVA *P* value < 10^−70^) (B) measured from cells grown in liquid or taken from swarms. Each distribution was calculated from more than 1,000 individual trajectories (500 min cumulative time) combined from three independent replicates. The behavioral response of fluorescent E. coli strain JLF394 was comparable to that shown by E. coli MG1655 (Fig. 2). (C and D) Representative fluorescence images of CheY-mYFP (C) and CheZ-mCherry (D) expression in single cells. The fluorescence signals form foci consistent with the expected cellular localization of CheY and CheZ. Mean, circle; median, square. See Materials and Methods for a description of JLF394. Download FIG S7, TIF file, 2.1 MB. Copyright © 2019 Partridge et al. 2019 Partridge et al. This content is distributed under the terms of the Creative Commons Attribution 4.0 International license.

In summary, our results support the idea that CheZ levels are elevated post-transcriptionally in swarmers by increasing the protein stability. This increase is consistent with the behavioral change in motility patterns during swarming.

### There is an optimal tumble bias that maximizes swarm expansion.

**FIG 7:**
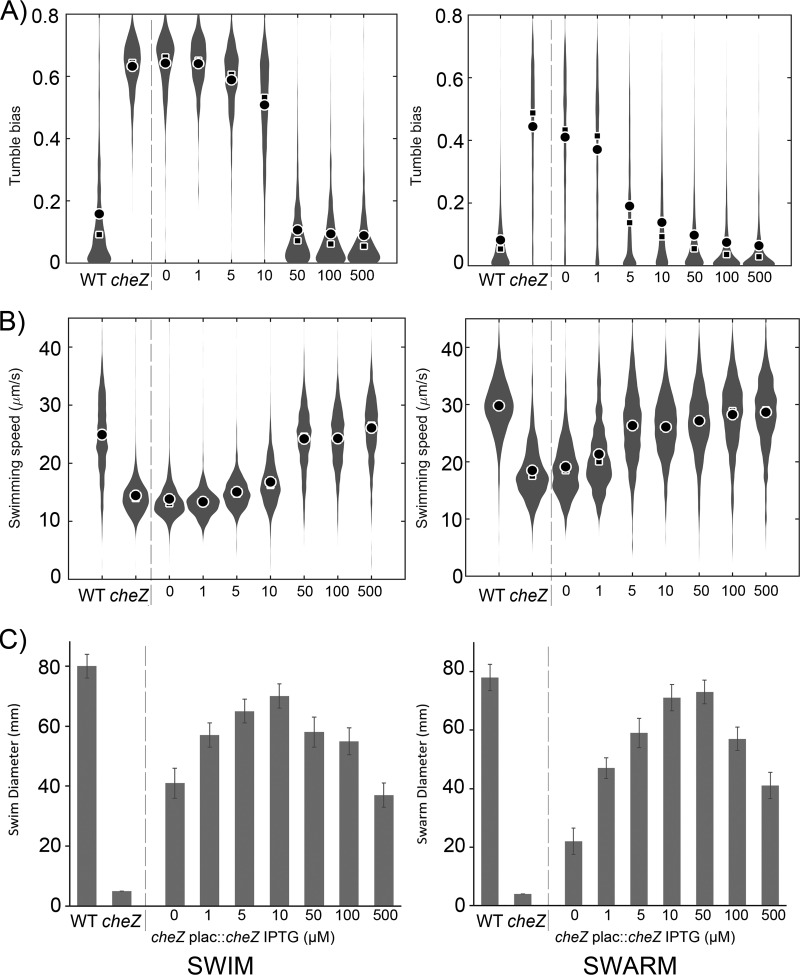
Optimal tumble bias for swarming. Tumble bias (A) and swimming speed (B) determined by tracking single cells of JP2716 (MG1655 Δ*cheZ* p*lac*::*cheZ*-Kan) as a response to increasing IPTG concentration. Controls included the MG1655 parent strain (WT) and its Δ*cheZ* derivative (separated from JP2716 with a dashed line). Each distribution was calculated from 1,600 to 5,000 individual trajectories (600 to 1,500 min cumulative time) combined from three independent experiments. Mean, circle; median, square. (C) The strains in panels A and B were tested for swimming (0.3% agar) (left) and swarming (0.5% swarm agar) (right) with the same range of IPTG concentrations. Plates were incubated at 30°C for 8 h. Data are representative of three biological cultures, each tested in triplicate. Error bars indicate standard deviations of the means.

With evidence showing that E. coli dampens its tumble bias during swarming, we asked if the observed low level of tumble bias is indeed the optimal level for swarming by modulating CheZ levels artificially. Initial attempts at CheZ expression from plasmid-borne promoters in a Δ*cheZ* mutant were fraught with issues of high levels of leaky expression. We therefore moved *cheZ* to place it under the control of the chromosomal *lac* promoter (JP2716; see Materials and Methods). CheZ levels were tunable above background with the gratuitous inducer IPTG (isopropyl-β-d-thiogalactopyranoside) at concentrations increasing continuously from 1 to 500 μM (Fig. S8). At each IPTG concentration tested, CheZ levels were higher in swarm cells than in swimming cells, in accordance with the increased stability of CheZ in the former (Fig. 6). To characterize the behavioral response of the strain to increasing levels of expression of CheZ, we tracked single cells from liquid cultures and swarm plates using the same range of IPTG concentrations (Fig. 7A and B). For each IPTG concentration, the observed tumble biases were on average lower in swarmers than in swimmers, consistent with the Western blot analysis. The tumble bias response is very sensitive to CheZ expression, especially in liquid cultures. In liquid, a tumble bias comparable to that seen with wild-type cells was achieved with an IPTG concentration of between 10 and 50 µM, while in the swarm agar, the wild-type tumble bias was matched at 50 µM IPTG. (The wild-type tumble bias of the swarmers indicated in Fig. 7A was slightly higher than that indicated for the swarmers in Fig. 2B, likely due the fact that these experiments were done by different people. The tumble bias difference between swimmers and swarmers remains significant). High IPTG concentrations brought the tumble bias of JP2716 lower than that of the wild type in both liquid and swarm agar. To determine which tumble bias maximizes performance for swimming and swarming, we measured the spread of colonies on swim plates (0.3% [wt/vol] agar) and swarm plates (0.5% [wt/vol] agar) using the same range of IPTG concentrations. The results yielded bell-shaped curves under both conditions, indicating that there is an optimal tumble bias (Fig. 7C). The maximum spreads of the colonies were seen at 10 µM in swim plates and 50 µM IPTG in swarm plates, supporting the hypothesis that a lower tumble bias improves performance for swarmers. In addition, maximum performance was achieved for the IPTG concentrations that yielded tumble biases that matched the wild-type tumble biases for each condition.

10.1128/mBio.00316-19.8 FIG S8 CheZ levels at increasing IPTG concentrations. JP2716 (MG1655 Δ*cheZ* p*lac*::*cheZ*-Kan) was propagated in liquid and swarm media supplemented with the indicated IPTG concentrations and analyzed in Western blots treated with CheZ antibodies as described in Materials and Methods. DnaK antibody was used as a loading control. Band intensities (calculated by Image J) were plotted as fold change with respect to the no-inducer liquid sample. A blot representative of three replicates is also shown. Download FIG S8, TIF file, 0.09 MB. Copyright © 2019 Partridge et al. 2019 Partridge et al. This content is distributed under the terms of the Creative Commons Attribution 4.0 International license.

Overall, artificial tuning of CheZ levels produced motility behavior consistent with an optimal range of tumble bias that is lower for swarming than for swimming. However, lowering the tumble bias further becomes detrimental for swarming.

## DISCUSSION

We show in this report that E. coli remodels its chemosensory physiology as a response to swarming. The altered physiology is likely the result of an elevation of the concentration of CheZ, which increases CheY∼P dephosphorylation bias and reduces the cells’ tumble bias. Reducing the tumble bias improves swarming performance, but a very low tumble bias negatively impacts expansion of the swarm, corroborating previous reports that tumbling is still necessary in the swarm (49). Therefore, there is an optimal tumble bias for swarming and E. coli is able to adapt its motility behavior to surface conditions that favor swarming.

The increased stability of CheZ in swarmer cells explains the increase in their CheZ levels (Fig. 6). However, the mechanism by which CheZ is specifically stabilized during swarming will require further investigation. In B. subtilis, contact with swarm agar increases synthesis of flagella by somehow sequestering a specific adaptor protein that otherwise (in liquid), in concert with the Lon protease, promotes degradation of a master regulator of biosynthesis of flagella; sequestration of the adaptor in swarmers results in 3-fold-to-10-fold-higher levels of the master flagellar transcription regulator, thus upregulating the entire flagellar regulon (50). This mechanism is unlikely to operate in E. coli, as transcription profiles of the chemotaxis genes do not change during swarming (see Fig. S6 in the supplemental material). However, bacteria in a swarm are known to sense the difference between swarm agar and hard agar and to alter the expression profiles of many genes (5). In *Salmonella*, for example, genes for iron metabolism, lipopolysaccharide synthesis, and type III secretion were induced only on swarm agar and not on hard agar. Although expression of the flagellar regulon is not altered under either surface condition, it is possible that other changes in the transcriptome or proteome of swarmers contribute to increased CheZ stability.

Why would a small change in CheZ concentration have a large effect on tumble bias? Goldbeter and Koshland (51) showed that when a kinase and phosphatase modify a substrate protein in a futile cycle (like CheA and CheZ on CheY), the steady-state concentration of the modified substrate (CheY~P) is ultrasensitive to small changes in the ratios of the two enzymes in working under saturation conditions, which is true for the chemotaxis system because the number of CheY molecules is much larger than the number of CheA and CheZ molecules (52). They called this phenomenon “zero-order ultrasensitivity.”

Why do swarmers alter the levels of CheZ, when tumble bias can be reduced in any number of other ways that alter CheA activity (Fig. 1)? CheA is part of a large complex, and it would be difficult to simply change CheA numbers. Changing CheB/CheR alters CheA activity but also changes the cell adaptation time. Thus, it appears that the bacteria have settled on the simplest solution, that of elevating CheZ levels, which would most directly reduce the steady-state concentration of CheY~P and is likely the most straightforward way for the cell to reduce tumble bias. We note that CheZ distribution in *Bacteria* is narrow and that CheZ is found mainly in proteobacteria (53). Among non-proteobacteria such as B. subtilis, other phosphatases (CheC/CheX) act on CheY (54). It would be interesting to see whether and how these bacteria alter their chemotactic program through their respective phosphatases during swarming.

How does a lower tumble bias benefit the swarm? An obvious advantage of increasing run lengths would be to maintain/promote the side-by-side alignment observed for packs or groups of cells moving within a swarm. The resulting cohesion imparted to the group would facilitate growth-fueled expansion of the swarm. We note that our measurements were recorded after transferring cells from the swarm to liquid. We expect that the tumble bias inside a dense swarm is likely suppressed entirely (16, 19) and is manifested mainly at the advancing edge of the swarm where cells are arranged in monolayers (5, 17, 23). While the lower tumble bias reduces chemotactic performance as measured by capillary assays (Fig. 5), the swarm is still proficient for chemotaxis as evidenced by the finding that a S. marcescens swarm (which also has a low tumble bias [1]) exhibits an avoidance response to antibiotics in the media (55). Thus, a functional chemotaxis system is apparently necessary for swarmers to steer clear of toxic chemicals. Whether a lower tumble bias facilitates expansion of the swarm by improving chemotactic performance on surfaces or through a nonchemotactic behavior still needs to be determined for E. coli, but other bacteria are proficient at chemotaxis toward particular chemicals while swarming (3).

Mutants in the chemotaxis genes cause a defective swarming phenotype in many bacteria. When cells are propagated on standard LB media, chemotaxis is not required *per se* for swarming in E. coli and *Salmonella* (12, 49, 56–58). Instead, a role for a basal tumble bias was observed to be important for hydrating the swarm colony (49, 57, 59), hydration being required for promoting flagellum-driven movement on a surface where there is little free water. How a basal tumble bias contributes to hydrating the swarm is unknown (6). Motor reversals are not only important for initiating swarming but also have been proposed to participate in continuously spreading the accumulated fluid within a swarm onto the virgin surface ahead, based on the observation that cells arriving at the edge of the swarm splay their flagellar filaments outwards during such events (17, 23). These maneuvers, as well as formation of forward-moving packs of cells augmented by a low tumble bias, could also spread the fluid outward by breaking through the surface tension at the colony edge. While the optimal tumble bias that the swarmers have arrived at likely includes all the functions listed above, we do not know whether within the various regions of the swarm (edge and interior) there is a specific distribution of cells with higher or lower biases for optimal organization and spreading of the swarm. Finally, Lévy walk (LW) behavior and patterns of collective vortices reported in swarms of S. marcescens and B. subtilis (19, 21, 60) likely emerge as a result of a low tumble bias. LW migration patterns are found throughout nature, including in large animals, and have been proposed to improve foraging when nutrients are scarce (61–64).

Swarmers show higher cell swimming speeds (Fig. 2), as well as higher motor speeds and torque (Fig. 4). The torque of the flagellar motor within a swarm is expected to be higher as a consequence of the higher external load exerted on the flagellar filament by surface viscosity or drag (65, 66). However, the higher torque was maintained during the bead assay, which was performed on tethered cells after several washing steps, even though the drag forces were identical for swimmer and swarmers in this assay (Fig. 4C). Therefore, the higher torque and speed that we observed for swarmer motors likely represent increased proton-motive force resulting from the altered swarmer physiology. For example, S. enterica swarmers are reported to upregulate tricarboxylic acid (TCA) cycle enzymes (42). A recent study measuring single-motor behavior of E. coli swarmers reported a CCW bias but not increased speeds (67). However, the speed measurements were performed on strains whose flagellar motors were locked in CW or CCW modes. Such strains do not swarm unless excess moisture is added to the surface (11, 57), so the cells were lifted from a wet-agar surface (67). It is not surprising that Ford et al. did not see a change in speed/torque because, as reported in our study, non-swarming cells taken from an agar surface do not show this behavior (Fig. 2).

In summary, E. coli has evolved a mechanism to lower its tumble bias for optimal swarming. By allowing more coherence in the group, a low tumble bias might enhance the ability of the swarm to expand its search for nutrients, independently of nutrient gradients. It is also possible that the altered chemosensory behavior contributes in some manner to the observed high tolerance of antibiotics, an emergent property of swarms (6, 55).

## MATERIALS AND METHODS

### Strains and growth conditions.

E. coli MG1655 was used as the wild-type strain for all experiments in this study. The *cheZ* deletion mutant was generated by P1 transduction of the *cheZ*::Kan insertion from JW1870 (The Coli Genetic Stock Center) into MG1655 before conversion to an unmarked deletion using the recombinase system of pCP20 (68), leaving a 82-to-85-nucleotide (nt) scar sequence in place of the cassette, to generate JP2523 (MG1655 Δ*cheZ*). A CheZ-expressing plasmid was generated by amplification of the *cheZ* coding sequence from MG1655 using appropriate primers incorporating NheI/SphI sites (JDP1146 and JDP1147) (see Table S1 in the supplemental material), digestion, and ligation into similarly cut sites in pBAD18-Kan (69) to give pJP379. This plasmid was used as a template to amplify a *cheZ*::Kan fragment using primers to incorporate regions flanking the coding region of *lacZ* (JDP1160 and JDP1147; Table S1). JP2523 was transformed with pKD46 and the amplified *cheZ*::Kan fragment following the protocol described by Datsenko and Wanner (68), to generate JP2716 (MG1655 Δ*cheZ* p*lac*::*cheZ*-Kan).

Cells were cultured in Lennox broth (LB; 10 g/liter tryptone, 5 g/liter yeast extract, 5 g/liter NaCl). Starting from single colonies isolated on agar plates, cells were grown to saturation overnight in broth cultures and were subcultured using a 1:100 dilution ratio in fresh medium and grown for around 4 h to an optical density at 600 nm (OD_600_) of 0.4. Liquid cultures were grown at 30°C in an Erlenmeyer flask on an orbital shaker at 200 rpm for aeration. Swarm agar and hard agar plates (LB solidified with 0.5% and 1.5% Eiken agar [Eiken Chemical Co., Japan], respectively) were poured and held at room temperature for 16 h prior to inoculation in the center with 6 µl of an overnight culture and incubated at 30°C. Swarm medium is normally supplemented with 0.5% glucose; therefore, the media for all tracking experiments (liquid as well as hard agar) were supplemented with 0.5% glucose to control for metabolic effects of glucose. For experiments performed with edge cells in a swarm, cells were collected after 4 h by gently washing the cells from the edge and were resuspended in various media for tracking assays (see below). Swim plates for measuring chemotaxis contained LB solidified with 0.3% Bacto agar.

### Time-lapse microscopy, cell tracking, and trajectory analysis.

Tracking experiments were carried out as described previously (31). Cells were harvested (2,000 × *g*, 5 min) and washed twice in fresh media. They were tracked at room temperature in LB or M9 minimal media supplemented with 0.05% (wt/vol) polyvinylpyrrolidone to prevent sticking of cells to the glass slide (31). Resuspended cells were diluted to an OD_600_ of approximately 0.01 to 0.05, and a 5-μl volume was introduced between a glass microscope slide and a 22-mm^2^ no. 1.5 coverslip, sealed using Valap sealant (consisting of equal amounts of petrolatum, lanolin, and paraffin wax). This created a channel that was ∼10-μm deep. Swimming cells were recorded at 10 frames per s with a digital scientific complementary metal-oxide semiconductor (CMOS) camera (Andor Zyla 4.2) (2-by-2 pixel binning, 50-ms exposure time, rolling shutter, full frame) mounted on an inverted microscope (Nikon Eclipse TI-U) with a 10× phase-contrast objective (Nikon CFI Plan Fluor; numerical aperture [NA], 0.30; working distance [WD], 16.0 mm) and LED white light diascopic illumination (Lumencor; PEKA light engine). The field of view was ∼1.3 mm square, containing 200 to 600 cells on average. A custom MATLAB (Mathworks) code was used to reconstruct the cell trajectories (github.com/dufourya/SwimTracker) (31) as follows. First, the mean pixel intensities of the frames from the entire image sequence were calculated to obtain an image of the background, which was subsequently subtracted from each image. The subpixel resolution coordinates of each cell in each frame were detected using a previously described method that uses radial symmetry (70) with an intensity detection threshold set to a false-discovery rate of 5%. Coordinates were linked to obtain cell trajectories using a previously described self-adaptive particle tracking method, u-track 2.1 (71), with motion model linkage cost matrices that combine constant velocity and random reorientation with an expected particle velocity of 20 to 30 μm/s and, otherwise, default parameters. Trajectories shorter than 5 s were discarded. Behavioral parameters such as speed and tumble bias were extracted from single-cell trajectories as previously described (31). The swimming speed was calculated by taking the average velocity of individual cells over their respective trajectories while excluding the frames where cells were predicted to be tumbling. To determine if the means of the swimming speed and tumble bias data were significantly different between treatment or strains, we performed a one-way analysis of variance (ANOVA). Each cell trajectory was weighted in accordance with its length to obtain an accurate quantification of cell-to-cell variability in the population. Because the configuration of our data set does not strictly respect all the assumptions underlying the analytical solution for the F-distribution (mainly normality), we performed a permutation analysis to obtain the empirical distributions. Ultimately, the empirical distributions were in good agreement with the analytical solution (see Fig. S9 in the supplemental material).

10.1128/mBio.00316-19.9 FIG S9 Analytical and empirical F-distribution for the one-way ANOVA. The empirical distribution (gray bars) was generated by performing random permutations of cell trajectories between treatment groups 10,000 times and calculating the F-statistic. The empirical distribution matches the analytical probability density function (PDF) with *n* = 15,300 and K = 3. Download FIG S9, TIF file, 1.8 MB. Copyright © 2019 Partridge et al. 2019 Partridge et al. This content is distributed under the terms of the Creative Commons Attribution 4.0 International license.

### Measurement of single-flagellum motor rotation.

Single-motor rotation experiments were performed as described previously (41). Briefly, E. coli MG1655 Δ*fliC* cells expressing FliC*^st^* from plasmid pFD313 (72) were induced for FliC*^st^* expression with 10 µM IPTG. Cells from liquid or swarm plates (as described above) were harvested in potassium phosphate motility buffer (10 mM potassium phosphate buffer [pH 7.0], 0.1 mM EDTA [pH 7.0], 10 mM NaCl, 75 mM KCl), their filaments were sheared, and 40 µl of the cell suspension was loaded onto a “tunnel” slide created by mounting a polylysine-treated coverslip on a glass slide using double-sided tape. After incubation at room temperature for 10 min, the tunnel was gently washed 3 times with 40 µl of motility buffer to remove unattached cells. Attached cells were exposed to 40 µl of a 1:50 dilution of polystyrene beads (Polysciences, Warrington, PA) (0.75 µm in diameter). The mixture was incubated at room temperature for 10 min to allow the beads to attach to the flagellar filaments. The wash steps were repeated to remove unattached beads. From harvesting to recording, around 25 to 30 min had elapsed. High-speed videos of individual beads were captured, and bead rotation was used as an indicator of motor function. The rotational motions of the beads were observed by phase-contrast microscopy (BX53F; Olympus, Tokyo, Japan) and recorded with a high-speed charge-coupled device (CCD) camera (ICL-B0620M-KC0; Imperx, Boca Raton, FL) at 1,250 frames/s. Phase-contrast images of each bead were cropped to the proper pixel size (16 by 16 to 22 by 22 pixels) and converted to videos (.avi files) with XCAP image processing software (Epix Inc., Buffalo Grove, IL, USA). Contrast enhancing, to fix image brightness and reduce background noise, was performed using ImageJ software (http://rsb.info.nih.gov/ij/). Videos of each bead were processed using custom analytical programs within LabVIEW 2012 (National Instruments, Austin, TX), which were provided by Yuichi Inoue (Ishijima Lab, Tohoku University, Sendai, Japan). Program 1 fitted each video by a two-dimensional Gaussian function to determine the center of the bead (*x*,*y* coordinates). The data were stored as a text file (.txt) and used by Program 2 to calculate the angular (rotation) speed from the center of the bead. Torque output of motors was calculated as described previously (43).

### Capillary assays.

Capillary assays were performed as previously described (45). Cultures were grown as described above under liquid, swarm agar, or hard agar conditions. Cells were harvested from each condition and gently washed (5,000 rpm, 3 min) twice in chemotaxis buffer (CB; 10 mM d-lactate, 0.01 mM l-methionine, 0.1 mM EDTA) before final resuspension in CB at an OD_600_ of 0.1. Capillaries (Fisherbrand) (75 mm in length, 1.2 mm in diameter) were melted shut at one end and then mildly heated by passage through a Bunsen flame before immersion in the attractant solution to draw in the solution, consisting of either CB alone or CB supplemented with 0.01, 0.1, 0.5, or 1 mM aspartate. Prepared capillaries contained comparable amounts of solution. Filled capillaries were dropped into Eppendorf tubes containing 500 µl of the relevant cell suspensions and incubated at 30 degrees for 30 min. After incubation, capillaries were removed from the cell suspension and their outer walls rinsed thoroughly before the sealed end was broken, allowing the intracapillary suspension to be expelled into a prechilled Eppendorf tube. The number of cells entering the capillaries was determined by plating dilutions of the capillary contents on LB agar and counting colonies after 24 h of incubation at 37°C.

### Western blots.

E. coli cells were cultivated under liquid or swarm agar and hard agar conditions. For surface growth, the “pour” method was used (34), where 5 ml of an exponential-phase culture was poured evenly onto the surface of a swarm/hard agar plate and poured off after 1 min. Plates were allowed to air-dry for 15 min before incubation. Cells were collected after 4 h growth and resuspended in phosphate-buffered saline (PBS) to an OD_600_ of 0.5. Whole cells were analyzed by SDS-PAGE and immunoblotting using appropriate antibodies to CheB or CheR (Ann Stock, Rutgers), CheY (Birgit Scharf, Virginia Tech), CheZ (Phil Matsumura, University of Illinois), and Tsr (John S. Parkinson, University of Utah), together with anti-DnaK (Abcam) as a loading/quantification control, using Amersham ECL Western blotting detection reagents (GE) according to the manufacturer’s instructions. Protein band intensities were quantified and compared using ImageJ software with a *t* test (utilizing the measured ratios) to determine *P* values from comparisons between the different conditions.

For monitoring CheZ stability, samples were prepared as described above, except that immediately after harvesting cells either from liquid or swarm conditions, 30 µM chloramphenicol was added, in excess of the 6 µM shown previously to inhibit translation (73). Cells were held at room temperature and sampled at various times by centrifugation, resuspension in SDS loading buffer, and flash-freezing. Samples were lysed by boiling prior to SDS-PAGE separation and immunoblotting performed as described above.

### RNA preparation and real-time PCR.

Bacteria were grown under swimming, swarm agar, and hard agar conditions as detailed above, supplemented with 0.5% glucose (wt/vol). Cultures (in triplicate) were harvested after 4 h of growth, standardized to an OD_600_ of 0.5, with 1 ml taken and mixed with twice the volume of RNAprotect reagent (Qiagen) before incubation was performed at room temperature for 5 min. Samples were pelleted at 12,000 rpm for 10 min before the pellet was used in total RNA extraction using a Qiagen RNeasy minikit with an On-column DNase digestion kit per the manufacturer’s instructions.

Real-time PCR (RT-PCR) was carried out using an iTaq Universal SYBR one-step kit (Bio-Rad) together with a ViiA-7 RT-PCR system (Thermo Fisher). Reactions were performed in duplicate, and the reaction mixtures contained 50 ng of total RNA, 10 µl iTaq green reaction mix (2×), 0.25 µl iScript reverse transcriptase, 150 nM (each) forward and reverse primers for relevant targets (Table S1), and nuclease-free H_2_O used to reach a final reaction volume of 20 µl. Threshold values were calculated using QuantStudio software (Thermo Fisher) and used to determine fold changes between samples. Student's *t* test was used to calculate *P* values. Data were normalized to the expression of *gyrA*.

### Fluorescent constructs and single-cell fluorescence quantification.

The construction of the fluorescent E. coli strain was performed as follows. First, gene fusions were constructed by assembling the following overlapping PCR products into a circular vector on the pUC19 plasmid backbone. The polypeptide linkers used to fuse CheY and CheZ to mYFP and mCherry were identical to linkers previously described as functional (48). pUC19 was linearized by PCR amplification using primers HS1 and HS2. *cheY* was amplified from E. coli MG1655 genomic DNA using primers HS3-2 and VIC1. *mYFP* was amplified from pYSD1003 (31) using primers VIC2 and HS10. *cheZ* was amplified from genomic DNA using primers HS11 and VIC60. *mCherry* was amplified from pYSD1005 (31) using primers VIC21 and HS12. The FLP recombination target (FRT)-kanamycin-FRT cassette was amplified from pCP15 (74) using primers HS5 and HS6-2. All the fragments were assembled in a single Gibson assembly reaction mixture following the standard protocol (75) to obtain plasmid pHSK007. The product was transformed by electroporation into E. coli DH10B and selected on an LB agar plate supplemented with 50 μg/ml kanamycin. The integrity of the construct was checked by sequencing. The cassette was amplified from pHSK007 by PCR using primers P-33 and P-35, and the linear DNA fragment was purified prior to genomic recombination. Sequences of all oligonucleotides used are shown in Table S1. Recombination of the cassette at the native locus in the genome of E. coli MG1655 to replace the wild-type copies of *cheY* and *cheZ* was done using the λ Red recombination protocol (68). E. coli MG1655 was first transformed with the pKD46 plasmid and then the purified linear cassette after induction of the recombinase from pKD46. Recombinant strains were selected on LB agar supplemented with 50 μg/ml kanamycin, and temperature-sensitive plasmid pKD46 was cured from the strain to obtain E. coli HSK249. The kanamycin resistance cassette was excised from the genome using flippase (Flp) by transforming plasmid pCP20 (74), leaving a single FRT sequence scar outside the *cheZ* coding sequence. Temperature-sensitive plasmid pCP20 was cured from the strain to obtain E. coli HSK311 (*cheY mYFP*, *cheZ mCherry*). Because HSK311 is poorly motile, the strain was passaged three times on 0.3% (wt/vol) agar plates with M9 salts supplemented with 1% (wt/vol) glycerol and 0.1% (wt/vol) Casamino Acids to select for mutants that had recovered wild-type motility performance. Cells from the edge of the colony were selected after 48 h of incubation at 32°C at each passage and inoculated onto a fresh plate. After the third passage, a mutant that had motility performance and growth rate equal to those of MG1655 was selected and named JLF394. Whole-genome sequencing revealed a region upstream of the master regulator *flhDC* to have been deleted (chromosome coordinates 1978502 to 1979279) encompassing an *insB1* insertional element as well as a binding site for LrhA, a negative regulator (76). This rearrangement is likely to cause upregulation of the *flhDC* promoter, consistent with the increased motility of JLF394.

Fluorescence microscopy images were acquired using an inverted microscope fitted with a 100× oil immersion objective (Nikon CFI Plan Fluor; NA, 1.30; WD, 0.2 mm), a solid-state white light source (Lumencor; Sola II SE), a digital scientific CMOS camera (Andor Zyla 4.2; 1-by-1 pixel binning, 1,000-ms exposure time, full frame, 16 bits). Cells were spotted on agarose pads (1% [wt/vol] agarose with M9 salts) after being washed twice in M9 salts and mounted between a glass slide and a no. 1.5 glass coverslip. Twenty different frames containing between 50 and 200 cells were acquired using phase-contrast mode and two fluorescence channels for each sample (for the YFP filters, excitation [ex] wavelengths, 500/20 nm; 515-nm longpass [LP]; emission [em] wavelengths, 535/30 nm; for the mCherry filters, ex wavelengths, 560/40 nm; 585-nm LP; em wavelengths, 630/75 nm). The camera dark current was subtracted from each image, and the uneven illumination was corrected using a flat-field image acquired using uniform fluorescent slides. Cell outlines were determined using SuperSegger (77) on the phase-contrast images using the default parameters provided for E. coli cells. Cells with sizes deviating from the population by more than 3 standard deviations (<5%) were discarded from the analysis. Single-cell fluorescence intensities were calculated by summing the fluorescence signals over each cell area and subtracting the background fluorescence intensity. The autofluorescence of wild-type cells (MG1655) in each channel was determined and subtracted from the fluorescence intensities of cell expressing the fluorescent reporters.
