# Supplementary figures and images for: Escherichia coli Remodels the Chemotaxis Pathway for Swarming
Source: mBio. 2019 Mar 19;10(2):e00316-19. doi: 10.1128/mBio.00316-19 (PMC6426603; doi:10.1128/mBio.00316-19)

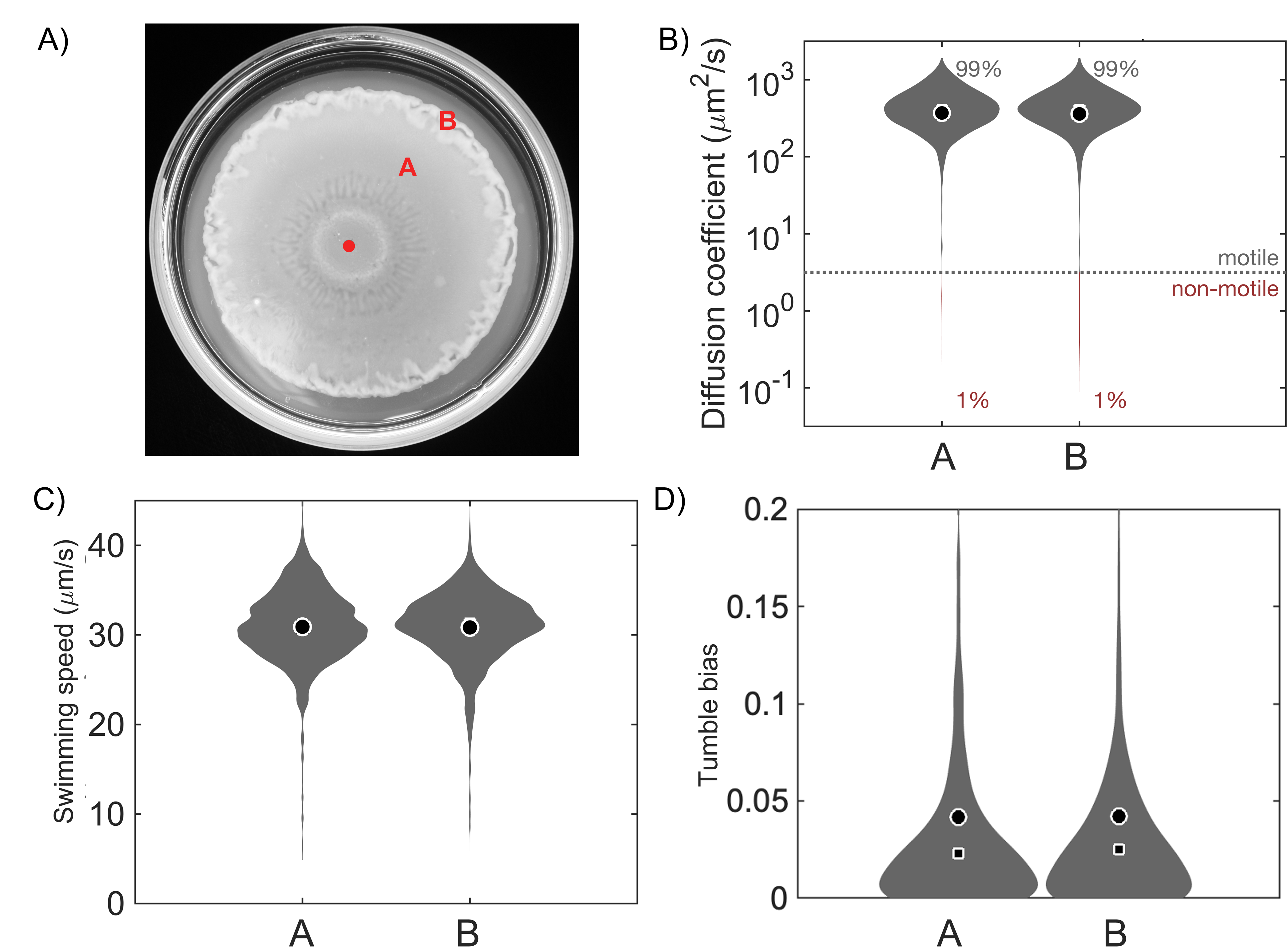

Supplement: FIG S1 [file mBio.00316-19-sf001.tif]

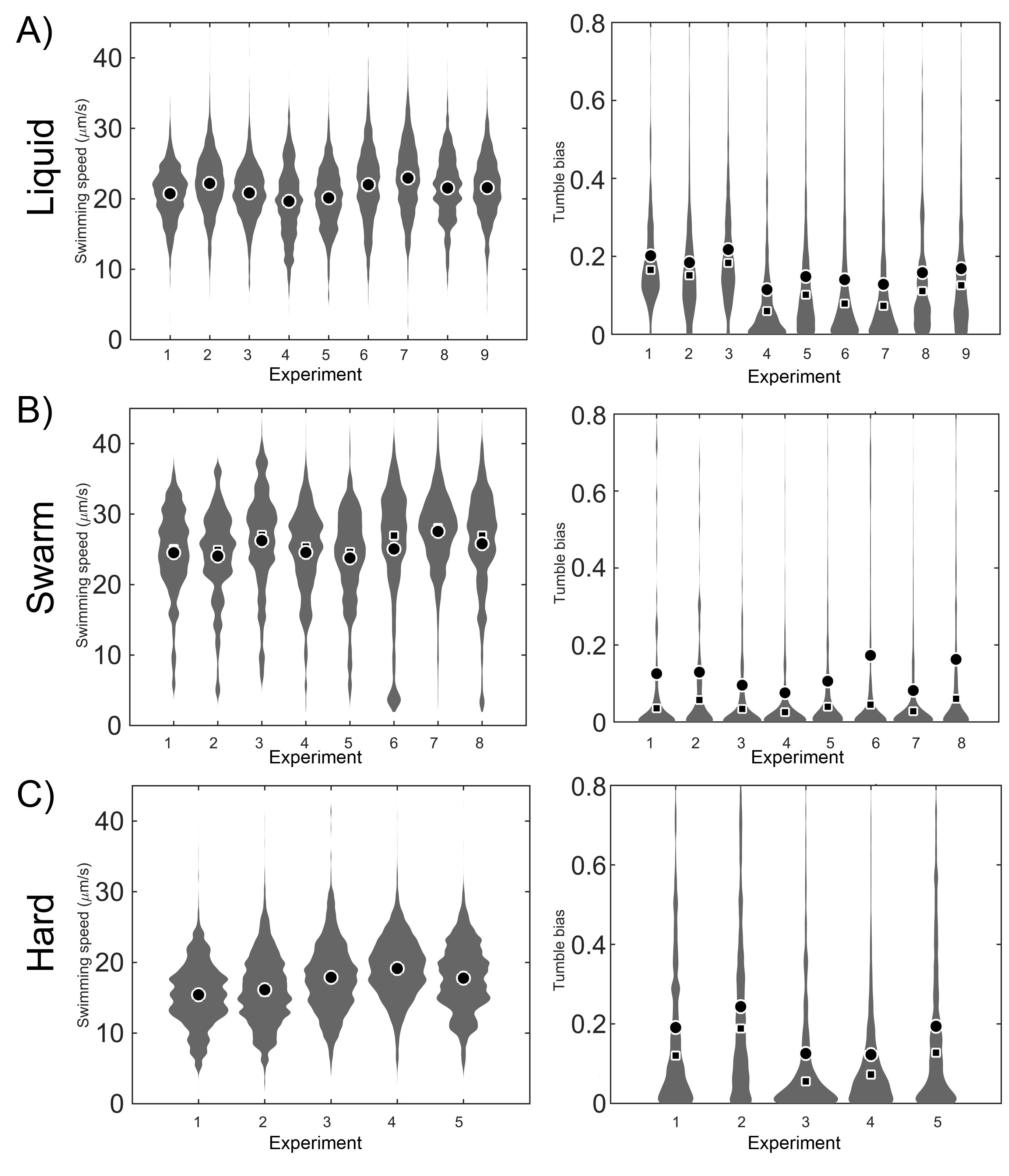

Supplement: FIG S2 [file mBio.00316-19-sf002.tif]

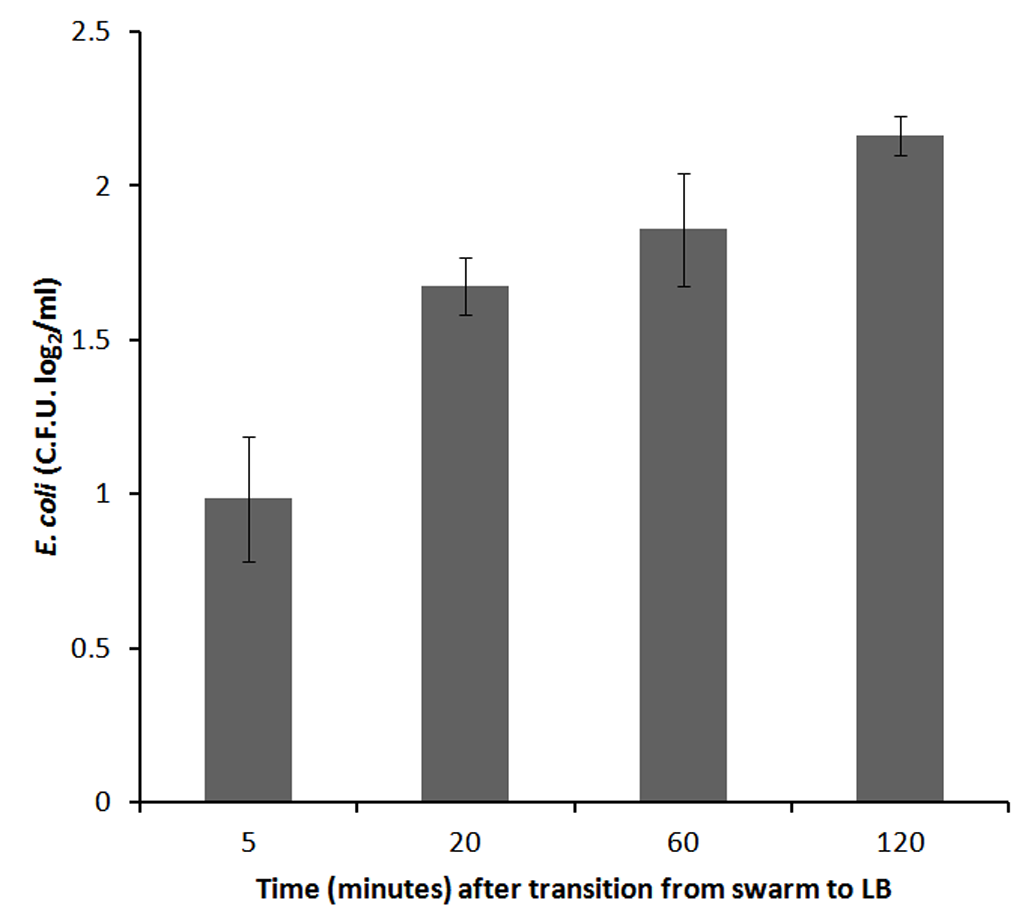

Supplement: FIG S3 [file mBio.00316-19-sf003.tif]

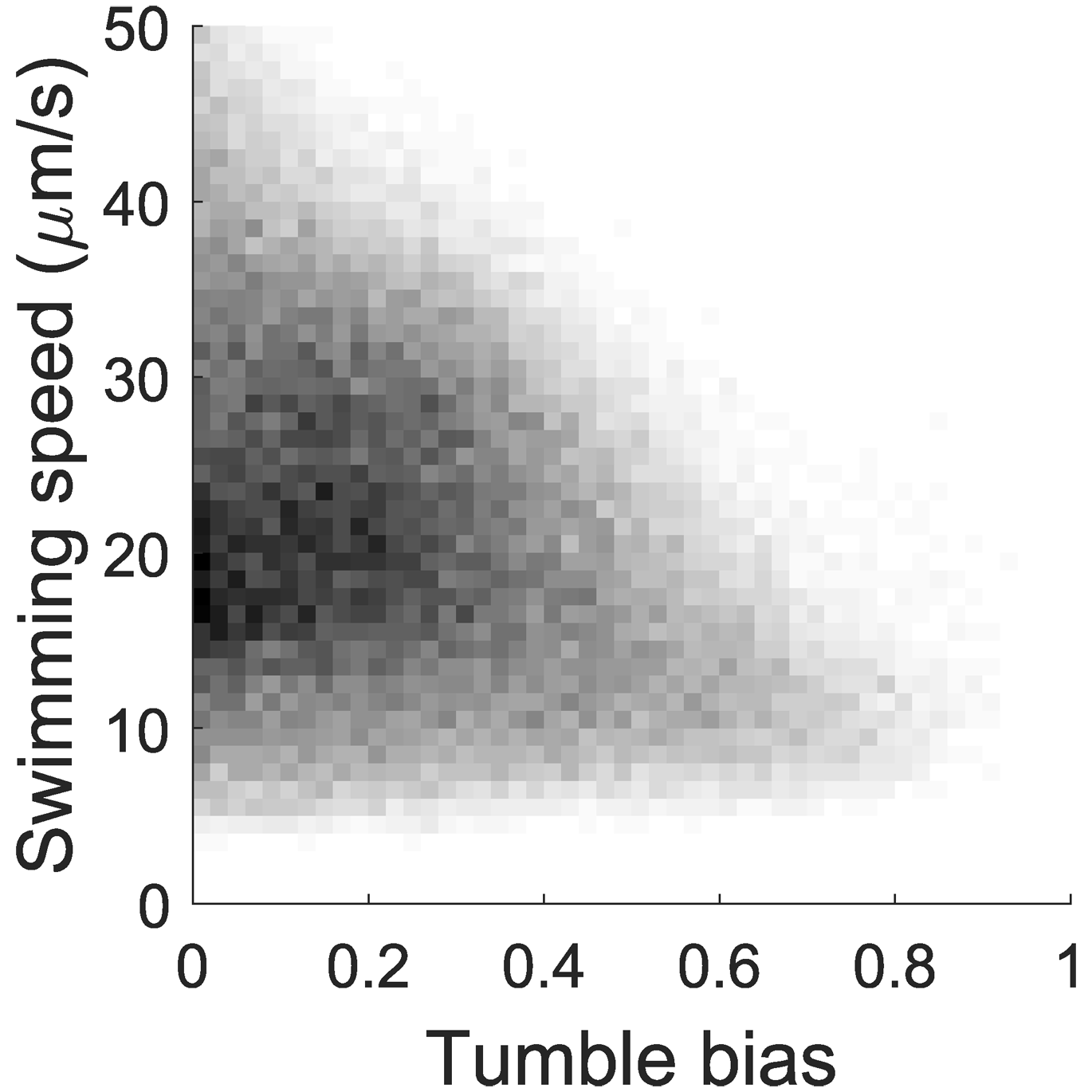

Supplement: FIG S4 [file mBio.00316-19-sf004.tif]

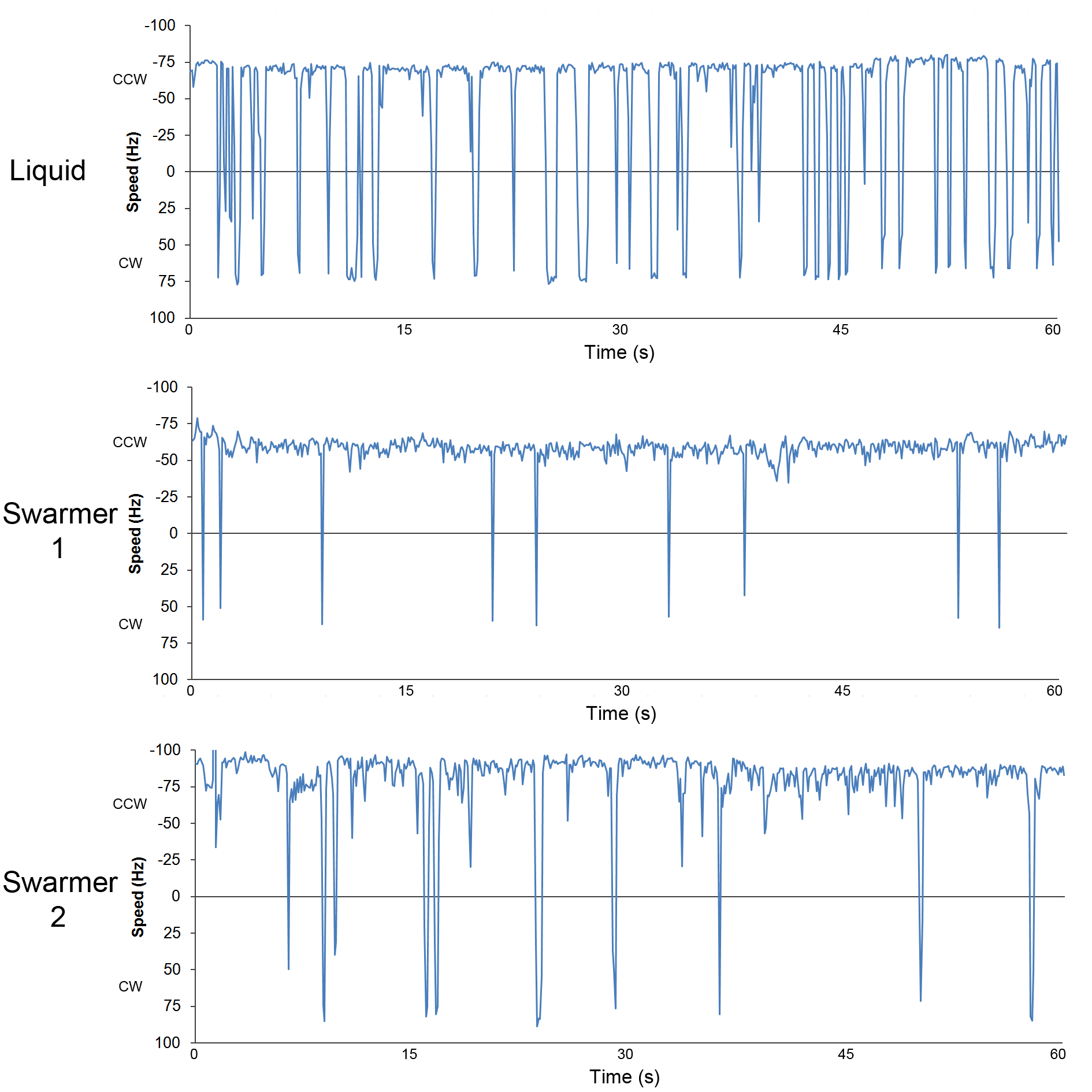

Supplement: FIG S5 [file mBio.00316-19-sf005.tif]

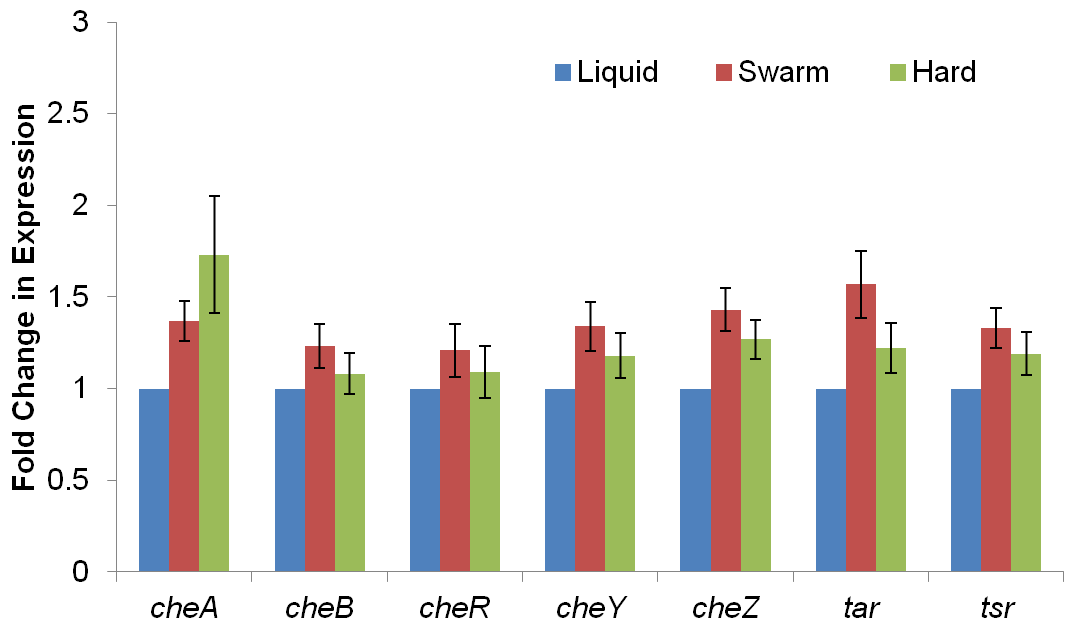

Supplement: FIG S6 [file mBio.00316-19-sf006.tif]

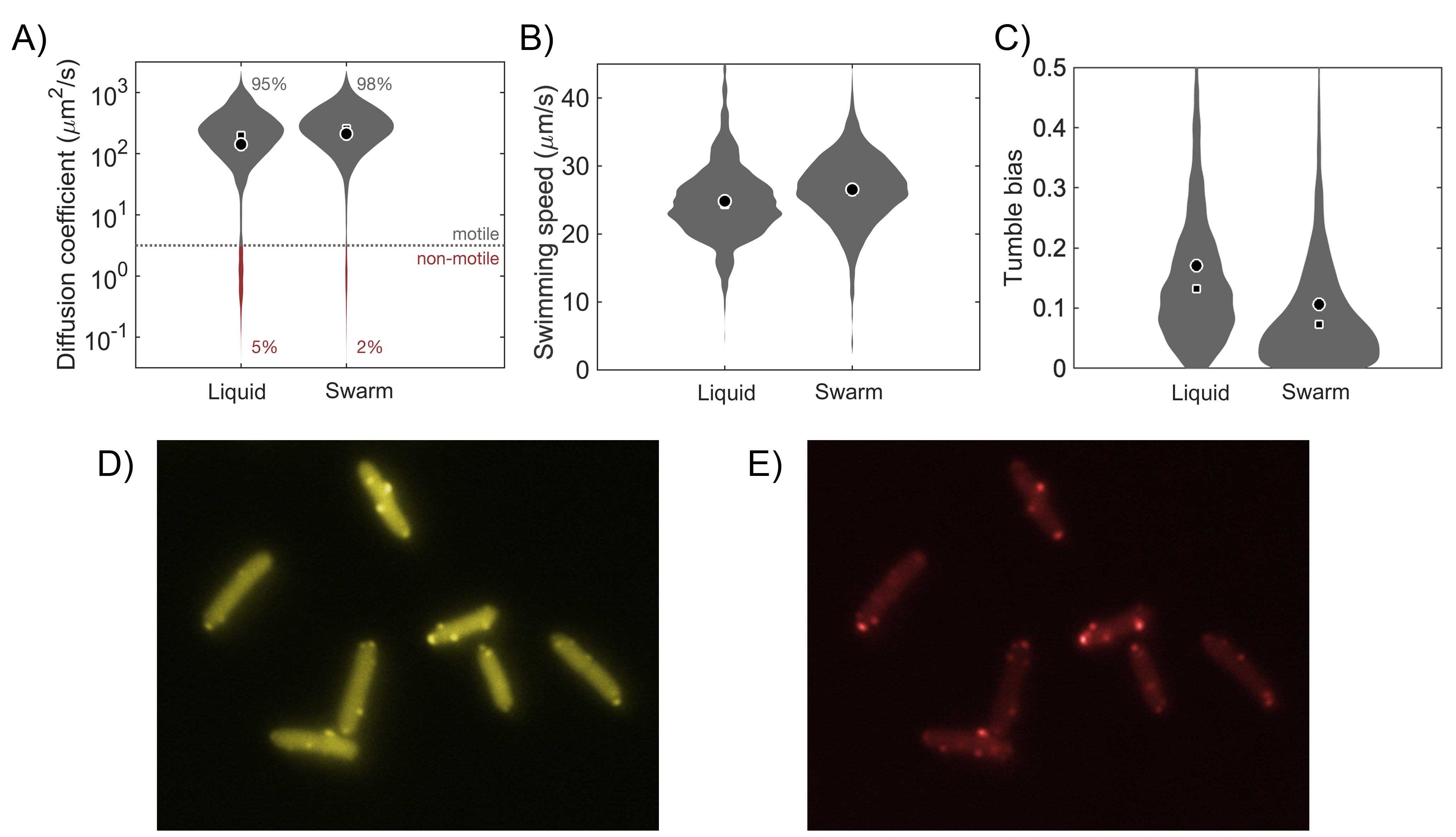

Supplement: FIG S7 [file mBio.00316-19-sf007.tif]

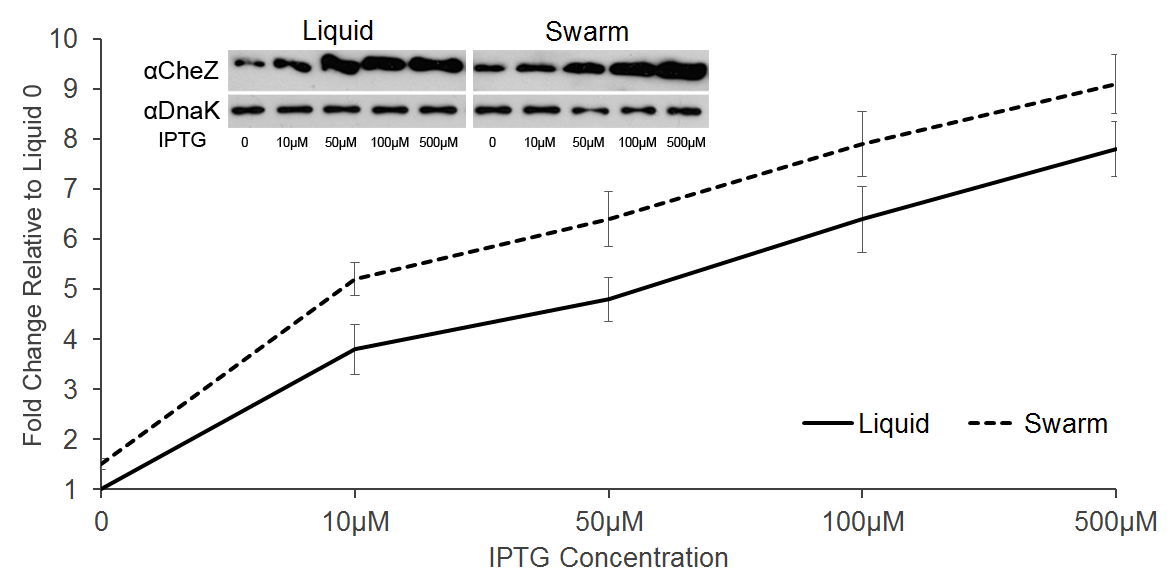

Supplement: FIG S8 [file mBio.00316-19-sf008.tif]

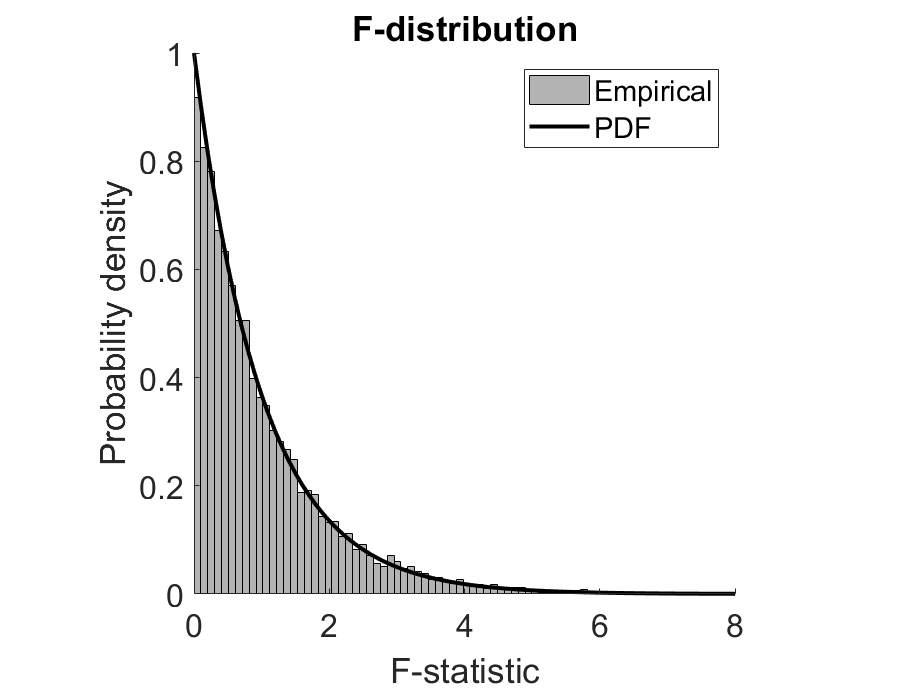

Supplement: FIG S9 [file mBio.00316-19-sf009.tif]
