# Supplementary material for: Escherichia coli Remodels the Chemotaxis Pathway for Swarming
Source: mBio. 2019 Mar 19;10(2):e00316-19. doi: 10.1128/mBio.00316-19 (PMC6426603; doi:10.1128/mBio.00316-19)
Supplement: TABLE S1 [file mBio.00316-19-st001.docx]

**Supplementary Table 1.**

|  | **Sequence** | **Target** |
| --- | --- | --- |
| JDP1060 | cgcgatatcgtcactgacaacgc | *tar* - forward |
| JDP1061 | ggtattcgccaggttaccaccg | *tar* - reverse |
| JDP1062 | cgcagcggcagagatcaaacg | *tsr* - forward |
| JDP1063 | ggcgatatcatggagccgatcg | *tsr* - reverse |
| JDP1064 | cagctcgacgcttataaacagtcgc | *cheA* - forward |
| JDP1065 | gataattcgtcgcggcgactgac | *cheA* - reverse |
| JDP1066 | catcaacagccatagcgacatgg | *cheB* - forward |
| JDP1067 | catcacaacgggcattggacg | *cheB* - reverse |
| JDP1068 | gtgcgtaacctgctgaaagagctg | *cheY* - forward |
| JDP1069 | caatgccgacatcgcgccatc | *cheY* - reverse |
| JDP1070 | cagcctgacgcgtatgctgc | *cheZ* - forward |
| JDP1071 | gatcctgatgcggttgtgacgc | *cheZ* - reverse |
| JDP1072 | catggtgactcggcggtctatg | *gyrA* - forward |
| JDP1073 | cgagatcggccatcagttcatggg | *gyrA* - reverse |
| JDP1074 | cgttctggctgaccataaacgcg | *cheR* - forward |
| JDP1075 | gagggaaatgatgtgcctcacgg | *cheR* - reverse |
| JDP1110 | tttccatggtgcaaccatcaatcaaacctgc | *cheZ* - forward w/ NcoI site |
| JDP1111 | tttaagctttcaaaatccaagactatccaacaaatcg | *cheZ* - reverse w/ HindIII site |
| HS1 | gatcctctagagtcgacctg | pUC19-rev |
| HS2 | cgggtaccgagctcgaattc | pUC19-for |
| HS3-2 | caggtcgactctagaggatcatggcggataaagaacttaaa | *cheY* - forward |
| HS5 | gaattcgagctcggtacccg | FRT-Km-FRT - forward |
| HS6-2 | gaattcgagctcggtacccgaagcttcaaaagcgctctga | FRT-Km-FRT - reverse |
| HS10 | agtcgcatcctcacttgtacagctcgtccatg | mYFP - reverse |
| HS11 | ctgtacaagtgaggatgcgactatgatgcaa | *cheZ* - forward |
| HS12 | cgggtaccgagctcgaattcgtttgtcggatgcggcgtcatcgccttatcagaccgcctgatatgacgtggtcacgccacatcaggcaatacaaattacttgtacagctcgtccatg | mCherry - reverse |
| P-33 | atacgtatttaaatcaggagtgtgaaatggcggataaagaacttaaa | *cheY* - forward |
| P-35 | gcctgatatgacgtggtcacgccacatcaggcaatacaaaggcgccattcgccattc | FRT-Km-FRT - reverse |
| VIC1 | gctcaccactcctccgccgcccagtttctcaaagattttg | *cheY - reverse* |
| VIC2 | ggcggaggagtggtgagcaagggcgaggag | mYFP - forward |
| VIC21 | ggaggcggaggcggagtggtgagcaagggcgaggag | mCherry - forward |
| IC60 | tccgcctccgcctccaaatccaagactatccaac | *cheZ* - reverse |
